# A direct comparison between AML1-ETO and ETO2-GLIS2 leukemia fusion proteins reveals context-dependent binding and regulation of target genes and opposite functions in cell differentiation

**DOI:** 10.3389/fcell.2022.992714

**Published:** 2022-09-07

**Authors:** Yi-Fan Zhang, Xiao-Lin Wang, Chun-Hui Xu, Na Liu, Ling Zhang, Yu-Ming Zhang, Yin-Yin Xie, Yuan-Liang Zhang, Qiu-Hua Huang, Lan Wang, Zhu Chen, Sai-Juan Chen, Robert G. Roeder, Shuhong Shen, Kai Xue, Xiao-Jian Sun

**Affiliations:** ^1^ Shanghai Institute of Hematology, State Key Laboratory of Medical Genomics, National Research Center for Translational Medicine at Shanghai, Ruijin Hospital Affiliated to Shanghai Jiao Tong University School of Medicine, Shanghai, China; ^2^ CAS Key Laboratory of Tissue Microenvironment and Tumor, Shanghai Institute of Nutrition and Health, Shanghai Institutes for Biological Sciences, Chinese Academy of Sciences, University of Chinese Academy of Sciences, Shanghai, China; ^3^ Laboratory of Biochemistry and Molecular Biology, The Rockefeller University, New York, NY, United States; ^4^ Key Laboratory of Pediatric Hematology and Oncology, Ministry of Health, Department of Pediatric Hematology and Oncology, Shanghai Children’s Medical Center, Shanghai Jiao Tong University School of Medicine, Shanghai, China

**Keywords:** AML1-ETO, ETO2-GLIS2, leukemia, transcription factor, transcriptional context, cell differentiation

## Abstract

The ETO-family transcriptional corepressors, including ETO, ETO2, and MTGR1, are all involved in leukemia-causing chromosomal translocations. In every case, an ETO-family corepressor acquires a DNA-binding domain (DBD) to form a typical transcription factor—the DBD binds to DNA, while the ETO moiety manifests transcriptional activity. A directly comparative study of these “homologous” fusion transcription factors may clarify their similarities and differences in regulating transcription and leukemogenesis. Here, we performed a side-by-side comparison between AML1-ETO and ETO2-GLIS2, the most common fusion proteins in M2-and M7-subtypes of acute myeloid leukemia, respectively, by inducible expression of them in U937 leukemia cells. We found that, although AML1-ETO and ETO2-GLIS2 can use their own DBDs to bind DNA, they share a large proportion of genome-wide binding regions dependent on other cooperative transcription factors, including the ETS-, bZIP- and bHLH-family proteins. AML1-ETO acts as either transcriptional repressor or activator, whereas ETO2-GLIS2 mainly acts as activator. The repressor-versus-activator functions of AML1-ETO might be determined by the abundance of cooperative transcription factors/cofactors on the target genes. Importantly, AML1-ETO and ETO2-GLIS2 differentially regulate key transcription factors in myeloid differentiation including PU.1 and C/EBPβ. Consequently, AML1-ETO inhibits, but ETO2-GLIS2 facilitates, myeloid differentiation of U937 cells. This function of ETO2-GLIS2 is reminiscent of a similar effect of MLL-AF9 as previously reported. Taken together, this directly comparative study between AML1-ETO and ETO2-GLIS2 in the same cellular context provides insights into context-dependent transcription regulatory mechanisms that may underlie how these seemingly “homologous” fusion transcription factors exert distinct functions to drive different subtypes of leukemia.

## Introduction

In the classical model of gene transcription, binding of a single transcription factor to a target gene is sufficient to recruit cofactors and RNA polymerases to initiate transcription, and the physical property of this transcription factor can largely determine the activation versus repression transcriptional states. However, in fact, most (if not all) genes are regulated by an array of multiple transcription factors, which cooperatively bind to the regulatory DNA elements and thereby set a unique transcriptional context (Ptashne and Gann, 1997; Spitz and Furlong, 2012). The activity of a transcription factor could be affected by the transcriptional context, including its position relative to other factors bound to the same DNA element and/or the abundance of these transcriptional factors and cofactors (Fry and Farnham, 1999). Indeed, elegant experiments with well-defined cellular models have shown that, even binding to the same target gene that is engineered with only few exchanged/rearranged transcription factor binding sites, a transcription factor could vary across repressive to active functions (Stampfel et al., 2015). Therefore, it is important to understand the function and mechanism of a transcription factor in a definable transcriptional context, and a robust cellular model will be helpful for achieving this goal.

Transcription factors and cofactors are the most common targets of chromosome translocations in leukemia. The produced fusion transcription factors play vital roles in leukemogenesis, and they can usually determine leukemia subtypes by regulating cell differentiation (Look, 1997; Tenen et al., 1997; Scandura et al., 2002). Notably, the ETO-family transcriptional corepressors, including ETO (also known as RUNX1T1), ETO2 (also known as CBFA2T3) and MTGR1 (also known as CBFA2T2), are all involved in leukemia-causing chromosomal translocations (Miyoshi et al., 1993; Nucifora et al., 1993; Gamou et al., 1998; Guastadisegni et al., 2010; Gruber et al., 2012; Thiollier et al., 2012). In every case, an ETO-family member fuses with a transcription factor to acquire a sequence-specific DNA-binding domain. In this regard, one would expect that the DNA-binding domain could determine the localization of the fusion protein on specific target genes, whereas the ETO moiety could manifest transcriptional activities through mechanisms shared by all members of the ETO-family corepressors. However, it is unclear to what extent this notion accounts for the binding and regulation of target genes by these fusion proteins in the cells.

In this study, we sought to perform a side-by-side comparative study between AML1-ETO and ETO2-GLIS2. AML1-ETO is the product of t(8;21)(q22;q22), the first chromosomal translocation ever discovered (Rowley, 1973), and is mainly associated with the French-American-British (FAB) M2 subtype of acute myeloid leukemia (AML) (Licht, 2001; Peterson and Zhang, 2004). AML1-ETO is fused by the DNA-binding domain (Runt domain) of the hematopoietic transcription factor AML1 (also known as RUNX1) and almost entire ETO protein which contains 4 conserved domains (nervy homology regions 1-4, or NHR1-4) (Licht, 2001; Peterson and Zhang, 2004). ETO2-GLIS2 is produced by the newly identified inv(16)(p13.3;q24.3), which is the most common translocation in non-Down syndrome M7 subtype of AML (also known as acute megakaryoblastic leukemia, or AMKL) (Gruber et al., 2012; Thiollier et al., 2012). ETO2-GLIS2 consists of the NHR1-3 of ETO2 and the DNA-binding domain (containing 5 Kruppel-like zinc fingers) of the GLI-like transcription factor GLIS2.

Previous studies from our group and others have shown that AML1-ETO can oligomerize through its NHR2 domain and then nucleate a stable multi-protein complex, AETFC, mainly involving NHR1- and NHR2-meidated interactions with E proteins (including HEB, E2A, and E2-2) and other transcription factors and cofactors (Zhang et al., 2004; Liu et al., 2006; Sun et al., 2013; Liu et al., 2019; Zhang et al., 2019). Similarly, ETO2-GLIS2 also forms a protein complex, and its oligomerization through the NHR2 domain is functionally important as well (Thirant et al., 2017). In addition, AML1-ETO and ETO2-GLIS2 share other similar properties in their functions and mechanisms, including 1) their DNA-binding domains (Runt and GLIS zinc fingers, respectively) are required for leukemogenesis (Yan et al., 2009; Thirant et al., 2017), and 2) their binding to target genes has been shown to be facilitated by the ETS-family transcription factor ERG (Martens et al., 2012; Thirant et al., 2017). However, despite these similarities, the AML1-ETO and ETO2-GLIS2 associated leukemia are dramatically different in their symptoms and prognosis. In particular, the AML1-ETO associated M2 AML occurs in both children and adult with better prognosis (Marcucci et al., 2005; Faber et al., 2016), whereas the ETO2-GLIS2 associated M7 AML is found only in children with poor prognosis (Gruber et al., 2012; Thiollier et al., 2012; Masetti et al., 2013; de Rooij et al., 2017; Hara et al., 2017; Smith et al., 2020; Liu et al., 2022). It is unclear whether these differences are due to their distinct biophysical properties or just because of their differential expression in corresponding hematopoietic cell types. In this regard, a direct comparison between AML1-ETO and ETO2-GLIS2 in a same cellular context would be helpful to answer these questions.

Therefore, we designed to ectopically express AML1-ETO and ETO2-GLIS2 in the U937 leukemia cell line and perform ChIP-seq and RNA-seq assays for a comparative transcriptomic analysis, as well as accompanied cell functional studies. The U937 cell line is a human immature monoblast cell line that can be induced to differentiate into heterogenous populations of cells in monocyte and macrophage lineages (Minta and Pambrun, 1985). This cell line has been widely used for studying functions and mechanisms of many leukemogenic fusion proteins including AML1-ETO (Burel et al., 2001; Pabst et al., 2001; Alcalay et al., 2003), PML-RARα (Grignani et al., 1993; Testa et al., 1994; Ruthardt et al., 1997; Grignani et al., 1998), PLZF-RARα (Ruthardt et al., 1997; Grignani et al., 1998), CBFβ-MYH11 (Helbling et al., 2005), and MLL-AF9 (Caslini et al., 2000). Notably, while most of these fusion proteins tended to inhibit myeloid differentiation of U937 cells, MLL-AF9 was found to be able to induce differentiation (Caslini et al., 2000). This notion provides a proof-of-concept that, in the same cellular context, unique properties of these fusion proteins could be uncovered. Furthermore, several important regulatory mechanisms have originally been discovered with these cellular models. Therefore, it will be interesting to use this well-defined system to determine whether AML1-ETO and ETO2-GLIS2 could exert similar or different functions and mechanisms in regulating gene expression and cellular functions.

## Materials and methods

### Establishment of stable cell lines

The stable cell lines were constructed by transduction of U937 cells with a retroviral vector system and by selection of single clones. In brief, the N-terminal HA-tagged AML1-ETO and ETO2-GLIS2 cDNAs were subcloned into a pRetrox-Tet3G vector that was equipped with puromycin resistance. The retroviruses were produced by co-transfection of 293T cells with the pRetrox-Tet3G and the psPAX2 and pMD2.G helper vectors. U937 cells were infected with filtered virus-containing supernatant of the 293T cultures, in the presence of 8 μg/ml polybrene (Sigma, H9268), and centrifuged at 1200 × g, 37°C for 90 min. Infected cells were selected with 1 μM puromycin at 12–24 h after infection. The survived cells were then subjected to a limiting dilution method in 96-well plates to obtain single cell clones of stable cell lines. To avoid the leaky expression before induction, the stable cell lines were grown in RPMI 1640 medium (ThermoFisher Scientific, C11875500CP) supplemented with 10% Tet-free fetal bovine serum (Clontech, 631106).

### Immunoblot

Cells were washed with cold PBS for three times and then lysed in RIPA buffer (Beyotime Biotechnology, P0013D) with 1% protease inhibitor (MCE, HY-K0010) and phosphatase inhibitor (MedChemExpress, HY-K0023). Total protein level of each sample was quantified with the BCA protein assay kit (ThermoFisher Scientific, 23227), and 20 μg of total protein was loaded per well for SDS-PAGE electrophoresis and membrane transfer. An anti-HA rabbit monoclonal antibody (Cell Signaling Technology, 3724) was used to detect the HA-tagged AML1-ETO and ETO2-GLIS2 proteins, and an anti-β-Actin mouse monoclonal antibody (Yeasen Biotechnology, 30101ES10) were used to detect β-Actin as a loading control. HRP-conjugated anti-rabbit IgG (Cell Signaling Technology, 77074) and anti-mouse IgG (Cell Signaling Technology, 7076) were used as secondary antibodies.

### Cell viability analysis

Cell viability was determined with Cell Counting Kit-8 (CCK-8) (Yeasen Biotechnology, 40203ES92). 5,000 cells were inoculated in each well of 96-well plate and cultured for certain time at 37°C. 10 μl CCK-8 solution was added into each well and incubated for 2–4 h. The absorbance at 450 nm, which reflected the dehydrogenase activity in living cells, was determined with a microplate reader.

### Cell cycle and apoptosis analysis

For cell cycle analysis, the cells were washed with pre-chilled PBS and fixed with 70% ethanal at −20°C. The fixed cells were resuspended with PBS, treated with RNase, stained with propidium iodide (PI) (Yeasen Biotechnology, 40711ES10), and subjected to flow cytometry analysis with Phycoerythrin (PE) channel. Cell apoptosis was analyzed with the Annexin V-FITC/PI Apoptosis Detection Kit (Yeasen Biotechnology, 40302ES60). The cells were washed with pre-chilled PBS and resuspended with binding buffer. The Annexin V-FITC and PI Staining Solution were added and incubated with the cells at room temperature for 10–15 min, and the stained cells were diluted with binding buffer and subjected to flow cytometry analysis with the PE and FITC channels within 1 h.

### ChIP-seq

ChIP-seq analysis was performed as described previously (Liu et al., 2019). The established U937 stable cell lines #4 and #14 were treated with 5 μg/ml doxycycline to induce their expression of AML1-ETO and ETO2-GLIS2, respectively. The cells were crosslinked with 1% formaldehyde for 10 min and stopped by 125 mM glycine. After cell lysis and sonication, 30 μl of anti-HA magnetic beads (MCE, HY-K0201) were added into the cell lysates and rotated overnight at 4°C. The precipitated DNA were decrosslinked, purified with the MinElute PCR Purification Kit (QIAGEN), and subjected to library construction and sequencing with the Illumina systems. ChIP-seq reads were aligned to the human reference genome (version hg19) with Bowite2 (version 2.2.9). Number of reads and mapping rate are shown in Supplementary Table S1. ChIP-seq peaks over background, and comparison with the input samples, were identified with MACS2 (version 2.1.1). DeepTools (version 3.1.2) was used in the subsequent data integrative analysis and visualization (Ramirez et al., 2016). HOMER was used in analysis of transcription factor binding motifs (Heinz et al., 2010).

### RNA-seq

The U937 stable cell lines #4 and #14 that contain AML1-ETO and ETO2-GLIS2, respectively, as well as a control cell line containing an empty vector, were treated with 5 μg/ml doxycycline for 12 and 48 h and were subjected to RNA-seq analysis. RNA was extracted with the TRIzol Reagent (Fisher Scientific, 15596018). TruSeq RNA Sample Preparation Kit, version2 (Illumina) was used to generate libraries. Illumina NovaSeq 6000 was used for sequencing. Clean sequenced reads were aligned to hg38 by HISAT2 (2.0.5) with default parameters. Aligned SAM files were handled using samtools (1.7). Aligned reads were extracted by Htseq (0.9.1) with default parameters. Number of reads and mapping rate are shown in Supplementary Table S1. Fragments per kilobase of exon model per million mapped reads (FPKM) were calculated by R script on its definition. Gene set enrichment analysis (GSEA) was performed with the version 7.5.1 of the Molecular signature database (MisgDB) using weighted statistics (Subramanian et al., 2005).

### Quantitative reverse transcription PCR

TRIzol Reagent (Fisher Scientific, 15596018) was used to lysis U937 cells for isolating total RNA, and PrimeScript RT reagent kit with gDNA Eraser (TaKaRa, RR047A) was used to synthesize cDNA. TB Green Advantage qPCR premixes (TaKaRa, 639676) was used for qPCR reaction on an ABI Prism 7500 Sequence Detection system (Applied Biosystems). The sequences of primers were listed as following: *GAPDH* forward-GATTCCACCCATGGCAAAT; *GAPDH* reverse-GACAAGCTTCCCGTTCTCAG; *RHOU* forward-GCTACCCCACCGAGTACATC; *RHOU* reverseGGCTCACGACACTGAAGCA; *SPI1* forward-GTGCCCTATGACACGGATCTA; *SPI1* reverse-AGTCCCAGTAATGGTCGCTAT. The expression levels of the genes were normalized against the internal control *GAPDH*.

### Induced cell differentiation

Phorbol-12-myristate-13-acetate (PMA; also known as 12-O-tetradecanoylphorbol 13-acetate, or TPA) induced differentiation of U937 cells to monocyte/microphage lineages was performed as described previously (Minta and Pambrun, 1985; Burel et al., 2001). To investigate the effects of AML1-ETO and ETO2-GLIS2 on PMA-induced differentiation, the cells were first treated with 5 μg/ml doxycycline for 24 h and then induced with 65 nM PMA for 2 days. An FITC-conjugated anti-human CD11b antibody (BioLegend, 301330) was used to assess the cell differentiation. Flow cytometry analysis was performed on the BD LSRFortessa cell analyzer.

## Results

### Induced expression of AML1-ETO and ETO2-GLIS2 in U937 cells exert similar effects on cell growth, cell cycle, and apoptosis

**FIGURE 1:**
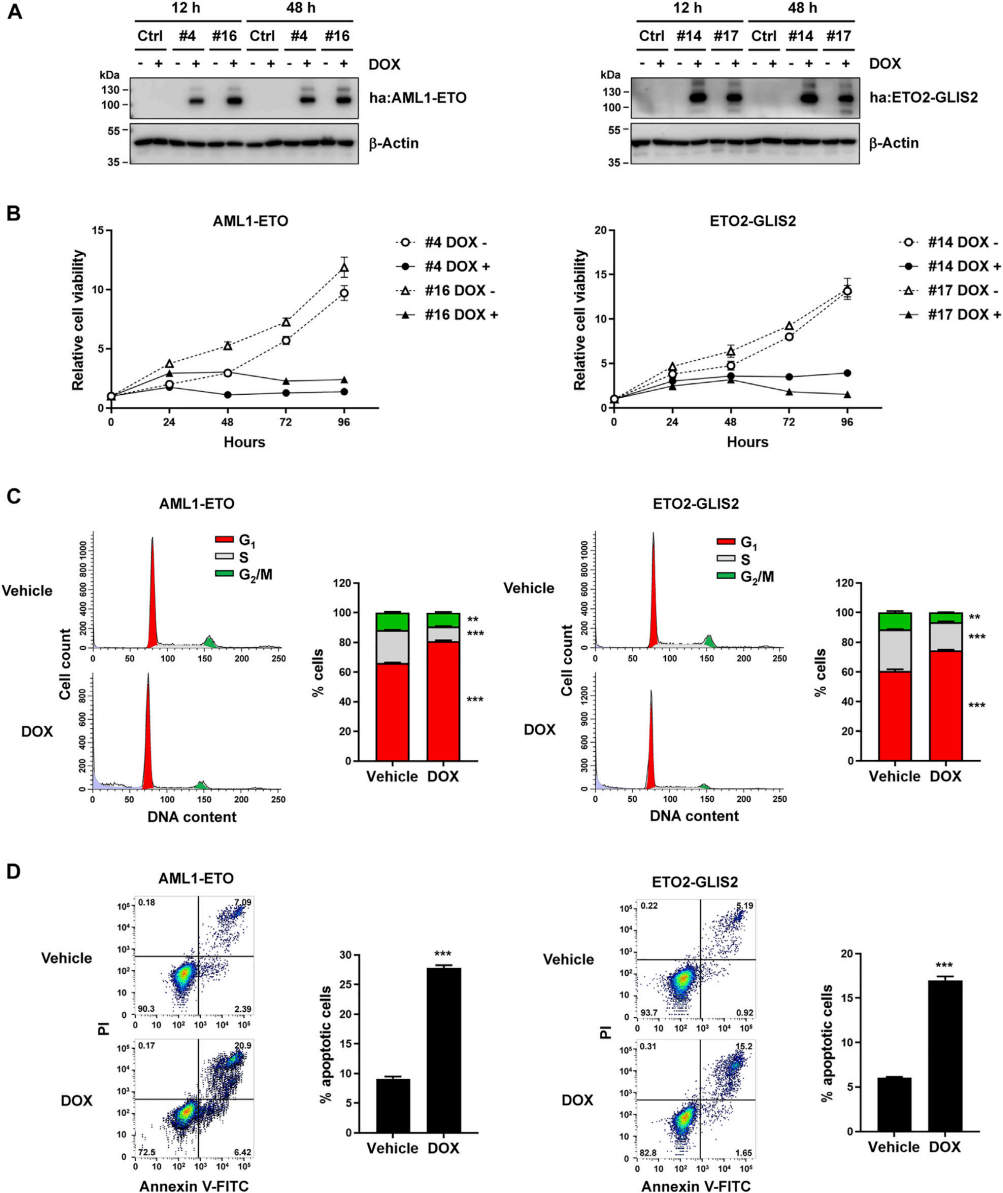
Establishment of the cellular models—induced expression of AML1-ETO and ETO2-GLIS2 in U937 cells showed similarly effects on cell growth, cell cycle, and apoptosis. **(A)** Immunoblot analysis of four stable cell lines (i.e., ^#^4 and ^#^16 for AML1-ETO and ^#^14 and ^#^17 for ETO2-GLIS2), showing their expression of the HA-tagged (ha) AML1-ETO and ETO2-GLIS2 induced by doxycycline (DOX). The analysis was performed for each cell line at 12 and 48 h after induction. The fusion proteins were detected with an anti-HA antibody, and β-Actin was used as a loading control. **(B)** Cell viability analysis of the indicated stable cell lines with and without doxycycline induction. **(C)** Cell cycle analysis of AML1-ETO (^#^4) and ETO2-GLIS2 (^#^14) cell lines with and without doxycycline induction. Each right panel shows the quantification and statistical analysis of the cells in G_1_, S, and G_2_/M stages in percentage. **(D)** Flow cytometry analysis of apoptotic cells using propidium iodide (PI) and Annexin V. Each right panel shows the quantification and statistical analysis. In panels **(B–D)**, data are presented as means ± SD of three separate experiments; two-tailed *t*-test; ***p < 0.001; **p < 0.01.

To directly compare AML1-ETO and ETO2-GLIS2 in the same cellular context, we used U937 cells to generate stable cell lines, in which the expression of AML1-ETO or ETO2-GLIS2 could be induced by adding doxycycline (DOX) in the cell culture medium. An HA-tag was inserted in the N-terminus of each protein to allow detecting and precipitating these proteins using the same protocol and condition. As a negative control, a stable cell line containing the empty vector was also generated. Immunoblot analysis validated that, in two AML1-ETO cell lines (#4 and #16) and two ETO2-GLIS2 cell lines (#14 and #17), the HA-tagged AML1-ETO and ETO2-GLIS2 proteins, respectively, could be properly expressed upon induction with doxycycline for 12 and 48 h (Figure 1A).

We then assessed the cellular behaviors upon induced expression of AML1-ETO and ETO2-GLIS2. Previous studies have shown that ectopic expression of several leukemogenic fusion proteins, including AML1-ETO, could inhibit cell growth and arrest cell cycle, probably involving mechanisms of oncogene-induced senescence (Pabst et al., 2001; Wajapeyee et al., 2010). Indeed, we observed that induced expression of AML1-ETO or ETO2-GLIS2 in U937 cells similarly inhibited cell growth, as indicated by the CCK-8 assays (Figure 1B). Flow cytometry-based cell cycle analysis showed that both AML1-ETO and ETO2-GLIS2 significantly increased the percentage of cells at G1 phase (Figure 1C), suggesting a blockage of the G_1_-to-S phase transition in the cell cycle. Furthermore, PI and Annexin V staining and flow cytometry analysis of the cells showed that the induced expression of AML1-ETO and ETO2-GLIS2 caused considerably increased numbers of apoptotic cells (Figure 1D). Thus, these results indicate that AML1-ETO and ETO2-GLIS2 exert comparable effects in U937 cells, and that these stable cell lines may serve as a useful tool for a comparative study of these two fusion transcription factors in the same cellular context.

### Although AML1-ETO and ETO2-GLIS2 can use their own DNA-binding domains to bind DNA, they share a large proportion of genome-wide binding regions dependent on other cooperative transcription factors

**FIGURE 2:**
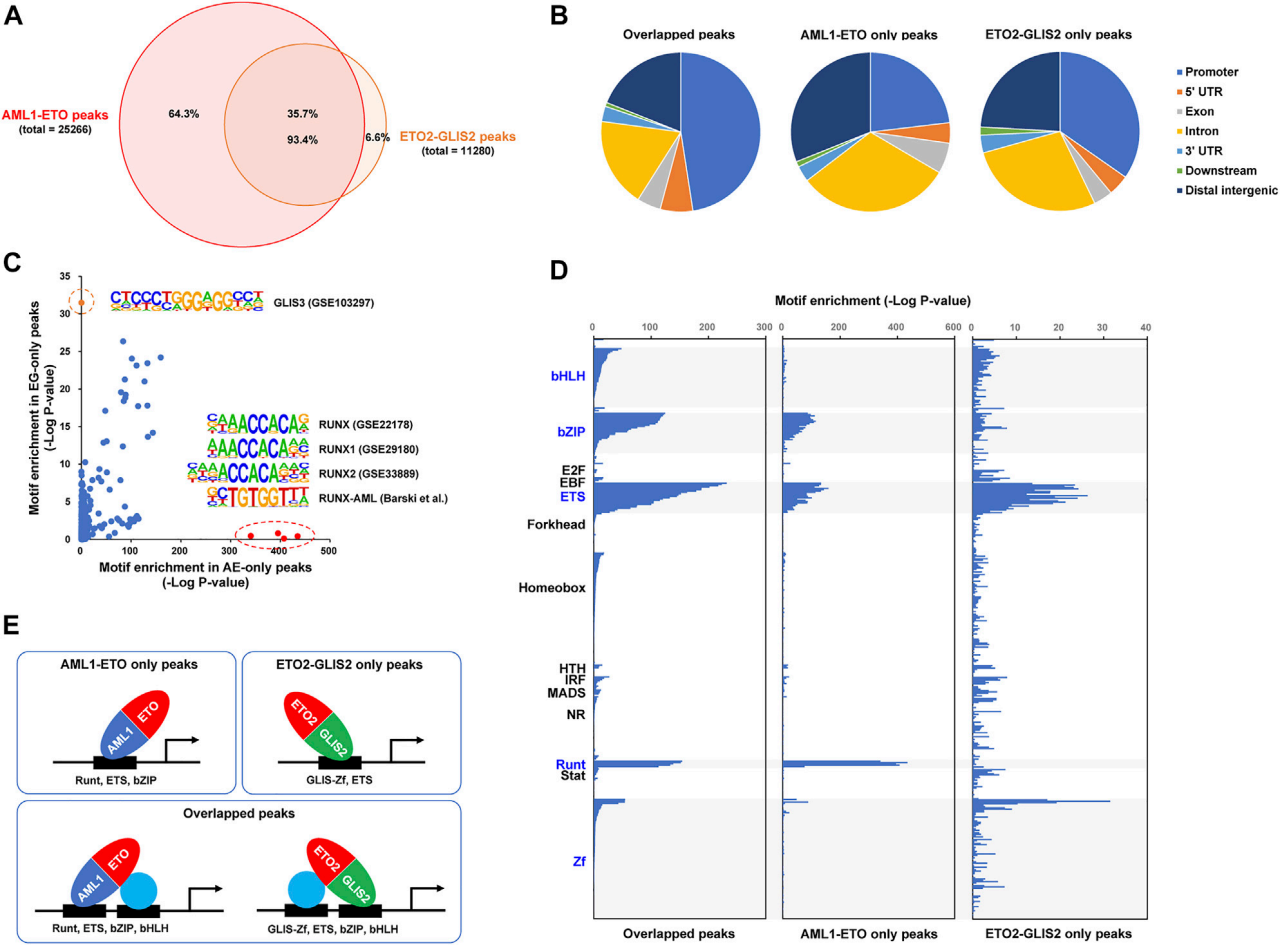
ChIP-seq analysis of how AML1-ETO and ETO2-GLIS2 bind to the genome. **(A)** Venn diagram showing total numbers of the peaks of AML1-ETO and ETO2-GLIS2 and their overlap. **(B)** Distribution of the overlapped and AML1-ETO and ETO2-GLIS2 only peaks in genomic regions related to gene structural and regulatory elements. **(C)** Enrichment analysis of transcription factor binding site (TFBS) motifs in the AML1-ETO and ETO2-GLIS2 only peaks. Circled are 4 Runt motifs and a GLIS3 motif that are specifically enriched in the AML1-ETO (AE) and ETO2-GLIS2 (EG) only peaks, respectively. **(D)** Comparison of the enriched TFBS motifs in the 3 classes of peaks. A total of 440 motifs are analyzed, and they are groups according to their DNA-binding domains (DBDs) and aligned along their enrichment score (minus log10 transformed *p*-value). The highlighted groups of motifs are those enriched in at least 1 class of the peaks. **(E)** A model depicting that, while AML1-ETO and ETO2-GLIS2 can use their own DBDs to bind DNA, they share large proportions of genome-wide binding regions dependent on the indicated cooperative transcription factors.

To investigate how AML1-ETO and ETO2-GLIS2 bind to DNA in the cells, we performed a ChIP-seq analysis of the doxycycline-induced cells (stable cell lines #4 and #14) with an anti-HA antibody under the same experimental conditions and peak calling algorithm. As a result, 25,266 peaks of AML1-ETO and 11,280 peaks of ETO2-GLIS2 were identified (Figure 2A). Manual inspection of the peaks on common target genes revealed that AML1-ETO and ETO2-GLIS2 have comparable levels of binding strength (see below for example genes), suggesting that the different numbers of peaks between AML1-ETO and ETO2-GLIS2 are not due to imbalanced non-specific binding signals. Notably, overlapping analysis of these peaks showed that the majority (93.4%) of the peaks of ETO2-GLIS2 are overlapping with AML1-ETO (Figure 2A). Such a high ratio of overlapped peaks is interesting, especially considering that AML1-ETO and ETO2-GLIS2 have completely different DNA-binding domains—the Runt domain binds to DNA with a consensus sequence motif TGT(C)GGT (Meyers et al., 1993), whereas the GLIS zinc finger (Zf) domain specifically recognizes a G-rich GLIS-binding site with a consensus sequence motif (G/C)TGGGGGGT(A/C) (Vasanth et al., 2011). Further analysis of genomic localizations of the overlapped, AML1-ETO only, and ETO2-GLIS2 only peaks showed that the overlapped peaks are more enriched in gene promoter regions, whereas the AML1-ETO only peaks are more enriched in the intergenic and intragenic regions (i.e., putative enhancers) (Figure 2B). This trend suggests potentially different biophysical properties of AML1-ETO and ETO2-GLIS2 (see below for further analysis).

We then performed a comprehensive analysis of the different classes of peaks for their enrichment of transcription factor binding motifs. In particular, four Runt motifs and a GLIS motif were found to be specifically enriched in the AML1-ETO only and ETO2-GLIS2 only peaks, respectively (Figure 2C). This result indicates that, at least in some genomic regions, AML1-ETO and ETO2-GLIS2 rely on their own DNA-binding domains to bind DNA. Such a clear correlation also provides a strong validation of our cellular model and experimental technologies.

Moreover, the results of a direct comparison of the motif enrichment patterns among the three classes of peaks revealed that 1) the ETS motifs are enriched in all three classes of peaks; 2) the bZIP motifs are enriched in the overlapped and the AML1-ETO only peaks; and 3) the bHLH motifs are enriched only in the overlapped peaks (Figure 2D). Based on these observations, we propose a model to explain how these different families of transcription factors cooperate with AML1-ETO and ETO2-GLIS2 to bind to the genome (Figure 2E). First, our results suggest that the ETS-family transcription factors play an important role in determining the binding regions of AML1-ETO and ETO2-GLIS2. Notably, this notion is consistent with previously reported association of ERG, a ETS family transcription factor, with both AML1-ETO (Martens et al., 2012) and ETO2-GLIS2 (Thirant et al., 2017) in the corresponding leukemia cells. However, because ERG is not expressed in U937 cells (data not shown), it is likely that the role of ETS family transcription factors in cooperating with AML1-ETO and ETO2-GLIS2 are not restricted to ERG, but can be similarly exerted by other family members, such as PU.1 or ELF1, which are relatively highly expressed in U937 cells (data not shown). Second, the enrichment of the bZIP motifs in the overlapped and the AML1-ETO only peaks suggests that the bZIP-family transcription factors, such as C/EBPα and C/EBPβ, may preferentially interact with the AML1 moiety of AML1-ETO (relative to the GLIS2 moiety of ETO2-GLIS2). This notion is supported by the well documented physical and functional interactions between C/EBPα and AML1-ETO (Westendorf et al., 1998; Pabst et al., 2001; Ptasinska et al., 2012; Loke et al., 2018; Ptasinska et al., 2019; Zhang et al., 2019). Third, the enrichment of the bHLH motifs selectively in the overlapped peaks suggests that the bHLH-family transcription factors associate with both AML1-ETO and ETO2-GLIS2 preferentially through interacting with their ETO moieties (but not their AML1 and GLIS2 moieties). This specificity is consistent with previous findings that several bHLH family transcription factors, including E2A, HEB, E2-2, and LYL1, form the AETC complex with AML1-ETO through interacting with the ETO moiety (Sun et al., 2013; Liu et al., 2019; Zhang et al., 2019). Similarly, it is strongly suggested that these bHLH-family transcription factors also interact with the ETO2 moiety of ETO2-GLIS2 through similar mechanisms, which may substantially contribute to the binding of ETO2-GLIS2 to the genome.

### AML1-ETO functions as transcriptional repressor and activator, whereas ETO2-GLIS2 mainly acts as an activator

**FIGURE 3:**
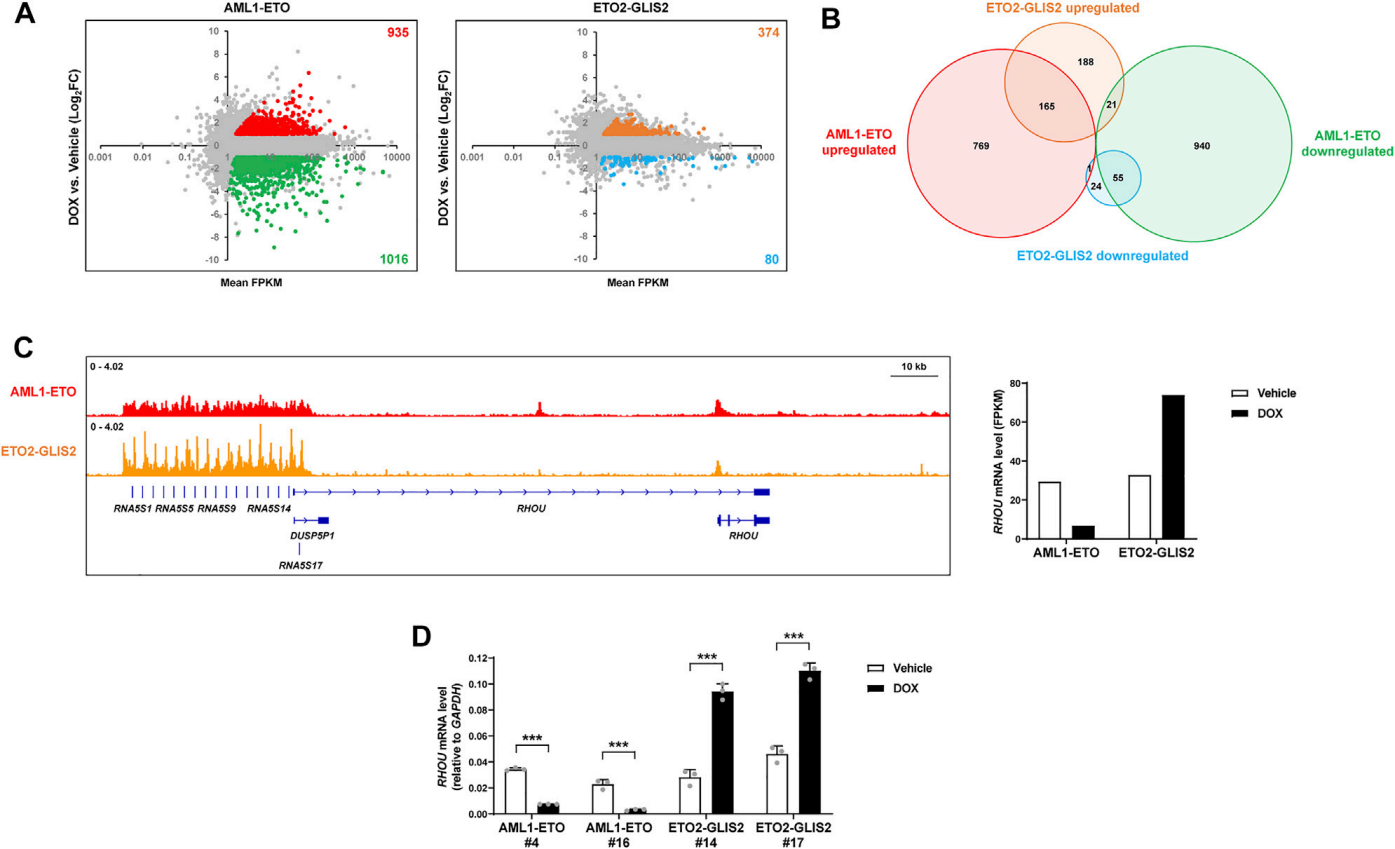
RNA-seq analysis of the AML1-ETO and ETO2-GLIS2 regulated genes. **(A)** Overviews of the expression levels of the genes regulated by AML1-ETO and ETO2-GLIS2. The dots for each upregulated and downregulated genes by AML1-ETO and ETO2-GLIS2 in more than 2-fold are labelled with different colors and counted. By using an empty vector-transduced control cell line as a negative control, the genes could be altered by DOX were excluded and labelled in gray. Mean FPKM of the samples with and without DOX induction was used to exclude the genes with very low levels of expression. **(B)** Venn diagram showing the numbers and relationships among the upregulated and downregulated genes by AML1-ETO and ETO2-GLIS2. **(C)** The *RHOU* gene as an example of the genes that are differentially regulated by AML1-ETO and ETO2-GLIS2. The ChIP-seq results show that they are both directly bound by AML1-ETO and ETO2-GLIS2, though with slightly but notably different binding patterns. Notably, the promoter regions of the tandem *RNA5S1*- *RNA5S17* genes (due to space limitation, listed in the figure do not include all the gene names) contain multiple G-rich GLIS binding motifs, therefore ETO2-GLIS2 shows repeated sharp peaks. **(D)** RT-qPCR analysis of the regulated expression of *RHOU* in independent clone of the cell lines expressing AML1-ETO (^#^4 and ^#^16) and ETO2-GLIS2 (^#^14 and ^#^17). Data are presented as means ± SD of three separate experiments; two-tailed *t*-test; ***p < 0.001.

To understand how AML1-ETO and ETO2-GLIS2 regulate the expression of target genes, we performed an RNA-seq analysis of the cells (stable cell lines #4 and #14) with and without doxycycline induction. We also used the empty vector control cell line in this analysis to exclude the genes that might be regulated by doxycycline treatment. As a result, we identified 935 upregulated genes and 1,016 downregulated genes by AML1-ETO (Figure 3A). This ratio of upregulated and downregulated genes is consistent with a previous profile of AML1-ETO target genes in U937 cells (Alcalay et al., 2003). In combination with many other studies performed in various systems, these results lead to the conclusion that AML1-ETO can function as both transcriptional repressor and activator. In contrast, the induced expression of ETO2-GLIS2 in U937 cells caused 374 upregulated genes and 80 downregulated genes (Figure 3A). Overlapping analysis of these regulated genes showed that about half of genes were not commonly regulated by AML1-ETO and ETO2-GLIS2, and some genes are even regulated in opposite directions (Figure 3B). These dramatic differences suggest that ETO2-GLIS2 may have a different transcriptional property and mainly act as an activator. Indeed, examination of the ChIP-seq results further revealed that, even for some genes (e.g., *RHOU*; Figure 3C) bond by both AML1-ETO and ETO2-GLIS2, these two fusion proteins function differently because they show slightly but notably different binding patterns and exert opposite activities in regulation of gene expression (Figures 3C,D).

### The activities of AML1-ETO as a repressor versus activator might be determined by the abundance of cooperative transcription factors/cofactors on target genes

**FIGURE 4:**
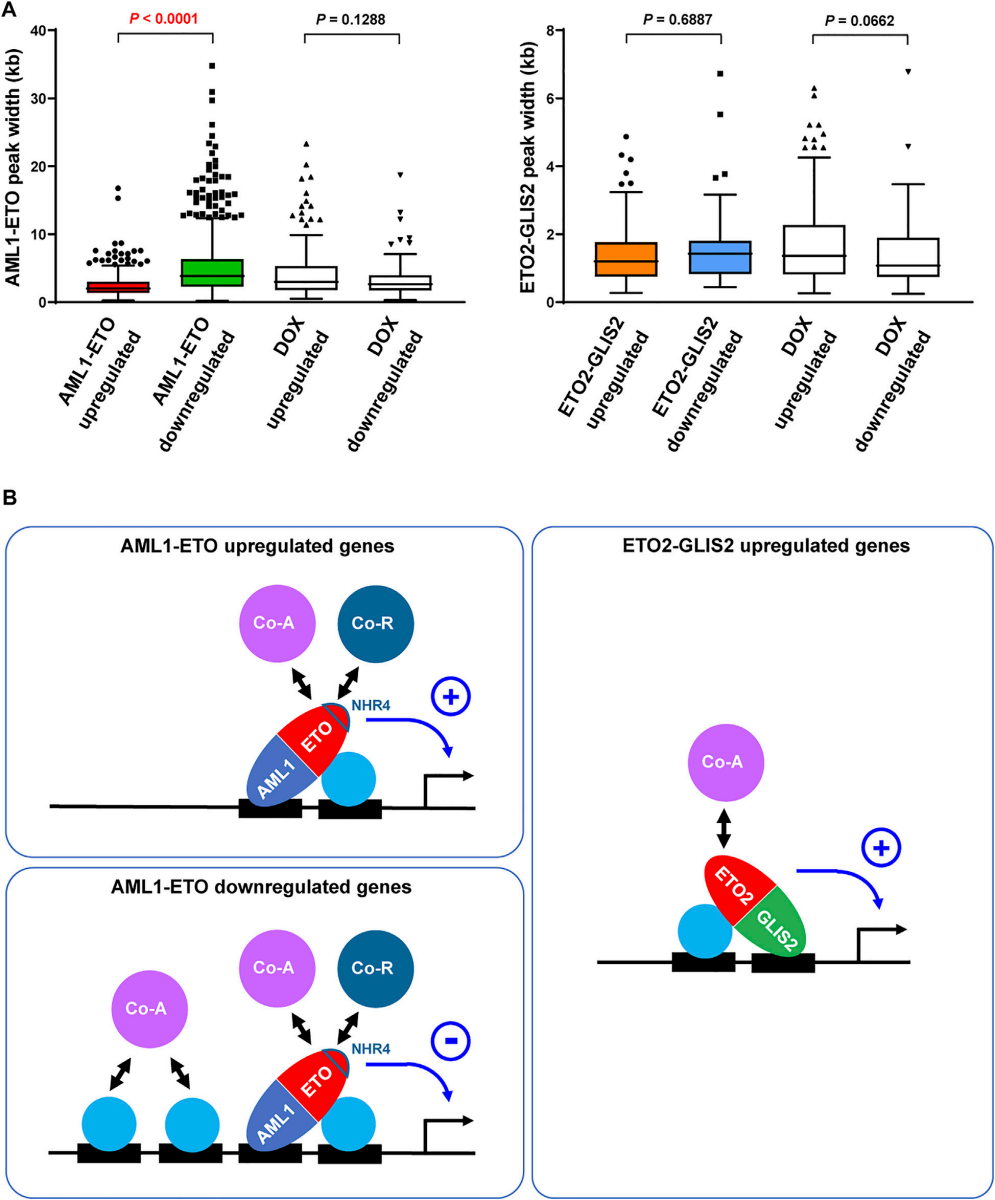
Possibly different functions and mechanisms of AML1-ETO and ETO2-GLIS2 in regulating target genes. **(A)** Significantly wider binding regions of AML1-ETO at the promoters of the downregulated relative to upregulated genes. This trend was not seen for the genes altered by DOX or for the ETO2-GLIS2 binding regions. **(B)** A working model explaining that the activities AML1-ETO as a repressor versus activator might be determined by the abundance of cooperative transcription factors and cofactors on target genes. In contrast, ETO2-GLIS2 mainly acts as a transcriptional activator. The AML1-ETO NHR4 domain, which is absent from ETO2-GLIS2, is highlighted because it may partially account for the different properties of these two fusion proteins. The circled plus and minus denote active and repressive activities, respectively. Co-A, coactivator; Co-R, corepressor.

To explore the mechanism of how AML1-ETO functions as either repressor or activator on different target genes, we reasoned that, as a transcription factor capable of recruiting both corepressor (Gelmetti et al., 1998; Lutterbach et al., 1998; Wang et al., 1998) and coactivator (Wang et al., 2011; Shia et al., 2012; Chen et al., 2015; Xu et al., 2019), AML1-ETO’s “net” activity on a target gene may be related to the “basal” transcription level conducted by the pre-existing transcription factors and cofactors. In this regard, we set out to compare the AML1-ETO upregulated and downregulated genes for the width of the AML1-ETO binding regions in their promoters, which may roughly reflect the abundance of transcription factors and cofactors in these regions (Kasper et al., 2010). As a result, we found that the AML1-ETO downregulated genes have wider AML1-ETO peaks in their promoters, compared with the AML1-ETO upregulated genes (Figure 4A). In contrast, this trend was not seen in the genes upregulated and downregulated by ETO2-GLIS2 or doxycycline for their AML1-ETO or ETO2-GLIS2 binding regions (Figure 4A). Therefore, it is possible that AML1-ETO exerts a transcriptional repressive activity on the transcription factor/cofactor-abundant genes, where the corepressors recruited by AML1-ETO may counteract the pre-existing coactivators to repress gene expression; conversely, on the transcription factor/cofactor-scarce genes, the coactivators recruited by AML1-ETO may have a chance to play a dominant role to activate gene expression (Figure 4B). In contrast, the ability of ETO2-GLIS2 to recruit corepressors may be limited (probably due to its lack of the NHR4 domain), providing a possible explanation for the observation that ETO2-GLIS2 mainly acts as an activator (Figure 4B)

### AML1-ETO and ETO2-GLIS2 differentially regulate key transcription factors that are important for myeloid differentiation

**FIGURE 5:**
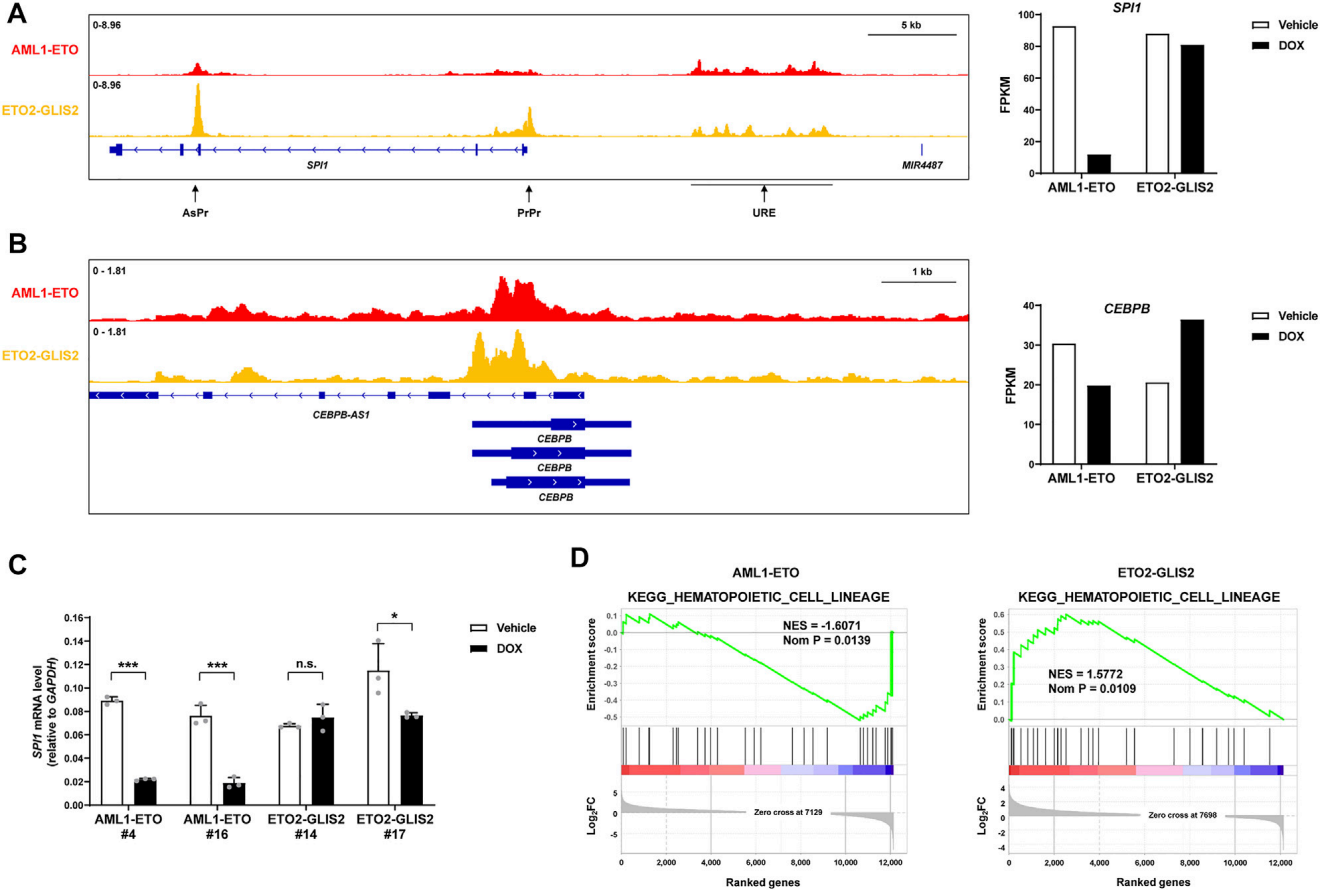
AML1-ETO and ETO2-GLIS2 differentially regulates key transcription factors that are important for myeloid differentiation. **(A)** *SPI1* (encoding PU.1) is repressed by AML1-ETO, but not ETO2-GLIS2. Three important regulatory elements of *SPI1*, the antisense promoter (AsPr), the proximal promoter (PrPr), and the upstream regulatory element (URE), are denoted with arrows. Note that AML1-ETO and ETO2-GLIS2 bind to URE at similar levels, whereas the levels of their binding to PrPr and AsPr are clearly different. **(B)** *CEBPB* (encoding C/EBPβ) is downregulated by AML1-ETO but upregulated by ETO2-GLIS2. **(C)** RT-qPCR analysis of the regulated expression of *SPI1* in independent clone of the cell lines expressing AML1-ETO (^#^4 and ^#^16) and ETO2-GLIS2 (^#^14 and ^#^17). Data are presented as means ± SD of three separate experiments; two-tailed *t*-test; ***p < 0.001; *p < 0.05; n.s., not significant. **(D)** Gene set enrichment analysis showing that the KEGG gene set of hematopoietic cell differentiation is negatively correlated with the AML1-ETO regulated genes, whereas it is positively correlated with the ETO2-GLIS2 regulated genes.

Transcription factors play central roles in cell differentiation. In monocyte/macrophage differentiation, the best-established transcription factors are PU.1 (encoded by the *SPI1* gene) and C/EBPβ (encoded by the *CEBPB* gene). Interestingly, we found that AML1-ETO and ETO2-GLIS2 both bind to, but differentially regulate these key transcription factor genes (Figures 5A,B). Previous studies (Trinh et al., 2021; van der Kouwe et al., 2021) have shown that the expression of *SPI1* is tightly regulated by three interplayed regulatory elements: an upstream regulatory element (URE) can directly interact with, and enhance the activities of, the conventional proximal promoter (PrPr) and an intragenic antisense promoter (AsPr); while PrPr drives the transcription of PU.1 coding mRNA, AsPr drives an antisense noncoding RNA, which may negatively regulate the PrPr activity; meanwhile, URE itself also drives a noncoding RNA termed *LOUP*, which can facilitate these enhancer-promoter interactions. Our ChIP-seq results revealed that AML1-ETO and ETO2-GLIS2 bind to URE at similar levels, but AML1-ETO has a much lower binding strength to PrPr and AsPr, compared with ETO2-GLIS2 (Figure 5A; left). Accordingly, the expression of *SPI1* is dramatically repressed by AML1-ETO, but not ETO2-GLIS2, as indicated by the RNA-seq results (Figure 5A; right) and RT-qPCR analysis of independent clones of both cell lines (Figure 5C). Therefore, in combination with previous studies (Trinh et al., 2021; van der Kouwe et al., 2021), these results suggest that AML1-ETO may interfere with interaction of URE with PrPr/AsPr probably through repressing *LOUP* transcription, subsequently leading to *SPI1* repression. In contrast, the binding of ETO2-GLIS2 to URE, PrPr and AdPr may not be able to interfere with these enhancer-promoter interactions, thus the expression of *SPI1* is not repressed. Similarly, both AML1-ETO and ETO-GLIS2 bind to the promoter of *CEBPB*, albeit with slightly different patterns, but their activities on the expression of *CEBPB* are opposite—AML1-ETO downregulates, but ETO2-GLIS2 upregulates *CEBPB* expression (Figure 5B).

The differential regulation of the key transcription factors by AML1-ETO and ETO-GLIS2 prompted us to speculate that the transcriptional programs of monocyte/macrophage differentiation might also be differentially regulated. To test this possibility, we performed a global transcriptome analysis of the RNA-seq data with GSEA. Indeed, the results showed that the gene set of hematopoietic cell differentiation is negatively correlated with the AML1-ETO regulated genes but positively correlated with the ETO2-GLIS2 regulated genes (Figure 5D), indicating that the transcriptional programs of the cells have been altered accordingly with the key transcription factors PU.1 and C/EBPβ.

### AML1-ETO inhibits, whereas ETO2-GLIS2 facilitates, myeloid differentiation of U937 cells

**FIGURE 6:**
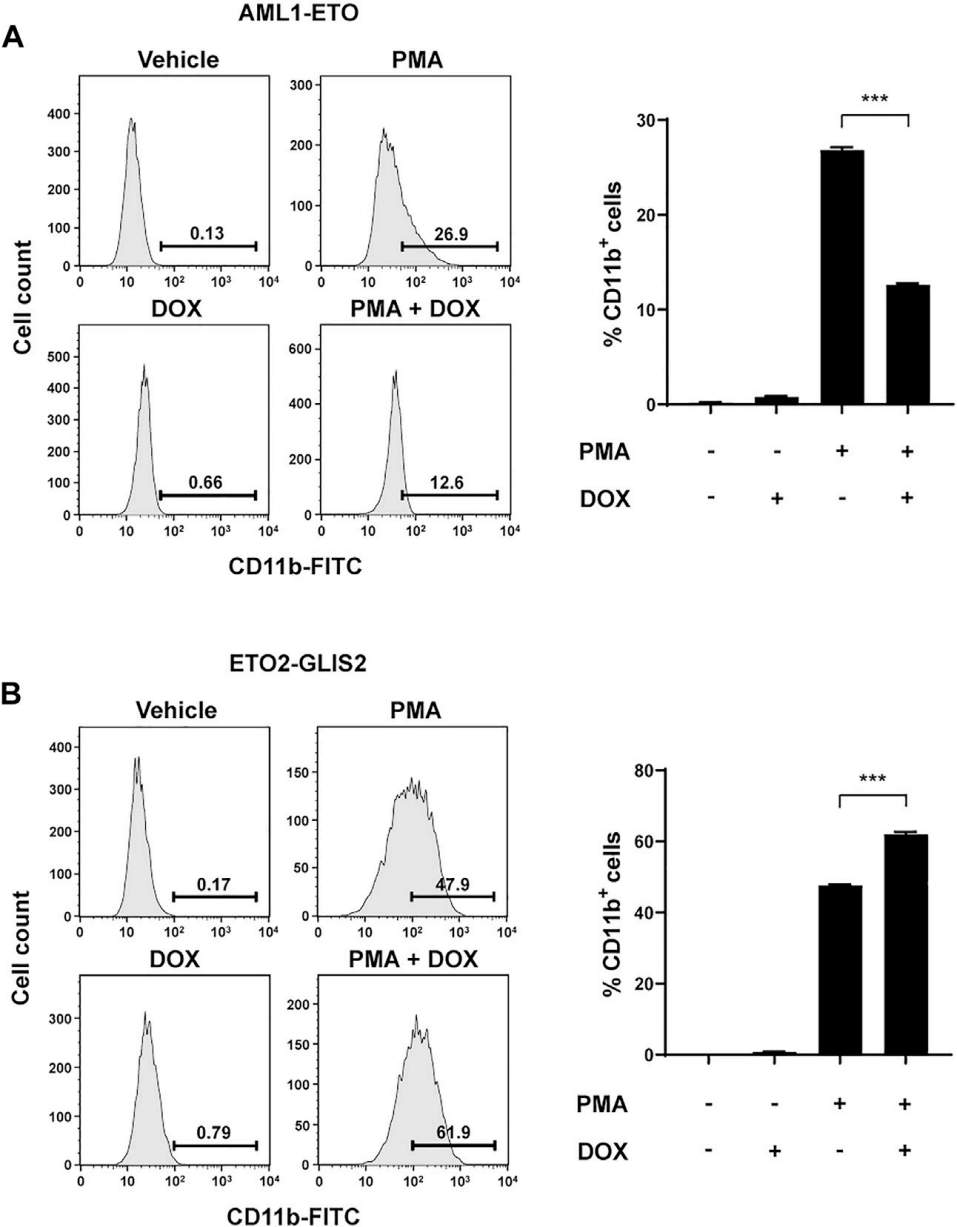
Opposite functions of AML1-ETO and ETO2-GLIS2 in regulating myeloid differentiation of U937 cells. **(A)** AML1-ETO inhibits myeloid differentiation of U937 cells. **(B)** ETO2-GLIS2 prompts this differentiation. Phorbol-12-myristate-13-acetate (PMA) was used as an inducer of differentiation of U937 cells to monocyte/macrophage lineages, and CD11b was used as a cell-surface marker. Left panel shows a representative flow cytometry result, and the right panel shows quantification and statistical analysis. Data are presented as means ± SD of three separate experiments; two-tailed *t*-test; ***p < 0.001.

To determine whether the altered transcriptional programs by AML1-ETO and ETO2-GLIS2 indeed affect the potential of cell differentiation, we used PMA to induce monocyte/macrophage differentiation of the U937 cells in the presence and absence of AML1-ETO or ETO2-GLIS2 in the cells. CD11b was used as a cell-surface marker for the differentiated cells. The results showed that the expression of AML1-ETO significantly inhibited the myeloid differentiation of U937 cells (Figure 6A), which is consistent with previous studies (Burel et al., 2001); in contrast, the expression of ETO2-GLIS2 significantly facilitated cell differentiation (Figure 6B). It is also notable that, even without doxycycline induction, the ETO2-GLIS2 cells showed higher percentage of CD11b^+^ cells (47.9%) than that of the AML1-ETO cells (26.9%) upon PMA treatment (Figures 6A,B). This effect was likely caused by leaky expression of these proteins in the cells. Nonetheless, induced expression of AML1-ETO and ETO2-GLIS2 could further decrease and increase the percentages of the CD11b^+^ cells, respectively (Figures 6A,B). Analysis of independent clones of the cell lines expressing AML1-ETO (#4 and #16) and ETO2-GLIS2 (#14 and #17) showed the same results (Supplementary Figure S1). Taken together, these results of molecular and cellular analyses in the same cellular context demonstrate that AML1-ETO and ETO2-GLIS2, despite their structural “homology” and widely shared target genes, can exert different, and even opposite, functions in regulating gene transcription and cell differentiation.

## Discussion

In this study, we performed a direct comparison between AML1-ETO and ETO2-GLIS2 in the same cellular context to clarify the similarities and differences between these two “homologous” fusion transcription factors. Functions and mechanisms of transcription factors are dependent on not only their own transcriptional properties but also the transcriptional context. In this regard, setting the same cellular context for the comparative study is helpful to reduce biological and experimental variances. Relative to analysis of each fusion protein in its corresponding leukemia cells, this is a complementary strategy that focuses more on the basic biophysical properties of these proteins, including how they bind to and regulate target genes. The U937 cells line was chosen for this study because 1) this immature monoblast cell line provides a proximate myeloid transcriptional context; 2) it maintains the potential of myeloid differentiation; and 3) it has been widely used for analyzing many leukemogenic fusion proteins, thus providing useful information for reference and comparison. Therefore, this well-defined U937 cell line serves as a type of “cellular test tube” for this comparative study. Using this system, we identified similarities and differences between AML1-ETO and ETO2-GLIS2 for their binding and regulation of target genes, as well as functions in regulating cell differentiation.

Our results clearly demonstrate that, although AML1-ETO and ETO2-GLIS2 can use their own DNA-binding domains to bind DNA, they share a large proportion of genome-wide binding regions. Further analysis suggests that the binding of AML1-ETO and ETO2-GLIS2 to the genome can be facilitated by several families of cooperative transcription factors. Notably, these cooperative transcription factors also show marked preference to different fusion proteins on certain binding regions, which is likely determined by the specific interaction mechanisms between the cooperative transcription factors and the fusion proteins through different domains/moieties. For example, the bHLH-family transcription factors E proteins specifically interacts with the ETO/ETO2 moieties of both AML1-ETO and ETO2-GLIS2, therefore these E proteins can determine some binding regions shared by AML1-ETO and ETO2-GLIS2, but not those bound exclusively by each. In contrast, as the bZIP-family transcription factors C/EBPα interacts with AML1-ETO through the AML1 moiety (Westendorf et al., 1998), it can preferentially facilitate AML1-ETO, but not ETO2-GLIS2, binding to the genome. Within the ETS family transcription factors, ERG has been shown to be physically and functionally associated with AML1-ETO and ETO2-GLIS2 in the corresponding leukemia cells; however, ERG is not expressed in U937 cells. In this situation, the ETS motifs are still highly enriched in all classes of binding regions (i.e., the shared and those bound exclusively by AML1-ETO and ETO2-GLIS2), suggesting that, except for ERG, other ETS-family transcription factors can similarly facilitate the genome-wide binding of AML1-ETO and ETO2-GLIS2. Interestingly, the absence of ERG from U937 cells and the replacement of ERG with other ETS-family transcription factors to associate with AML1-ETO and ETO2-GLIS2 may provide a mechanistic explanation for the cell-type specific functions of these fusion proteins. Furthermore, given the important role of ERG in self-renewal of hematopoietic stem cell (Lacadie and Zon, 2011; Taoudi et al., 2011), the absence of ERG in U937 cells may also explain why the roles of AML1-ETO and ETO2-GLIS2 in these cells are embodied as regulation of myeloid differentiation rather than self-renewal.

It is unexpected and interesting to find that AML1-ETO and ETO2-GLIS2 have opposite functions in regulating myeloid differentiation of U937 cells. Although the U937 cell line does not represent the appropriate stages of hematopoiesis when these fusion proteins occur or transform the cell to leukemia, the opposite effects of cell differentiation induced by AML1-ETO and ETO2-GLIS2 still reflect mechanistical differences in regulation of the genes essential for myeloid differentiation. The differential regulation of *SPI1* and *CEBPB* are of particular interest because they encode the most important transcription factors, PU.1 and C/EBPβ, respectively, in monocyte/macrophage differentiation. Because these analyses were performed side-by-side in the same cellular context and experimental conditions, it would not be difficult to use this system to further clarify the more detailed mechanisms, for example, by making mutations in the fusion proteins or in the regulatory elements of the target genes. Although being analyzed in the U937 cells, these regulatory mechanisms are unlikely restricted to U937 cells but may be applicable in broader myeloid differentiation and leukemogenic processes. Therefore, this type of “cellular test tube” may provide a useful tool to explore previously unknown mechanisms. Furthermore, because the U937 cell line has been widely used to study many leukemogenic fusion proteins, a retrospective comparison with previous studies would also be helpful to understand the new ones. For example, the differentiation promoting effect of ETO2-GLIS2 is strikingly reminiscent of a similar effect of MLL-AF9 observed previously (Caslini et al., 2000), which is rare among all the analyzed leukemogenic fusion proteins using this system. Therefore, it would be interesting to further compare between them (especially considering that both ETO2-GLIS2 and MLL-AF9 are associated with poor prognosis of leukemia patients) and to expand to other systems, for obtaining broader understanding of the mechanisms of leukemogenesis.

In summary, this study is the first direct comparison between AML1-ETO and ETO2-GLIS2 in the same cellular context. The results reveal the similarities and differences between them in their binding and regulation of target genes, as well as exerting cellular functions. In principle, the similarities enable them to be cooperated with the same families of other transcription factors and thereby share a large proportion of genome-wide binding regions, whereas the differences promote them to differentially regulate gene expression and cellular functions through transcriptional context-dependent regulatory mechanisms. The new mechanisms and insights may underlie how these seemingly “homologous” fusion transcription factors exert distinct biological functions and drive different subtypes of leukemia.

## Data Availability

The datasets presented in this study can be found in online repositories. The names of the repository/repositories and accession number(s) can be found below: ChIP-seq and RNA-seq data are available through the Gene Expression Omnibus (GEO) accession code GSE207218.
