# Supplementary material for: A direct comparison between AML1-ETO and ETO2-GLIS2 leukemia fusion proteins reveals context-dependent binding and regulation of target genes and opposite functions in cell differentiation
Source: Front Cell Dev Biol. 2022 Sep 7;10:992714. doi: 10.3389/fcell.2022.992714 (PMC9490184; doi:10.3389/fcell.2022.992714)
Supplement: Supplementary file 1 [file DataSheet1.PDF]

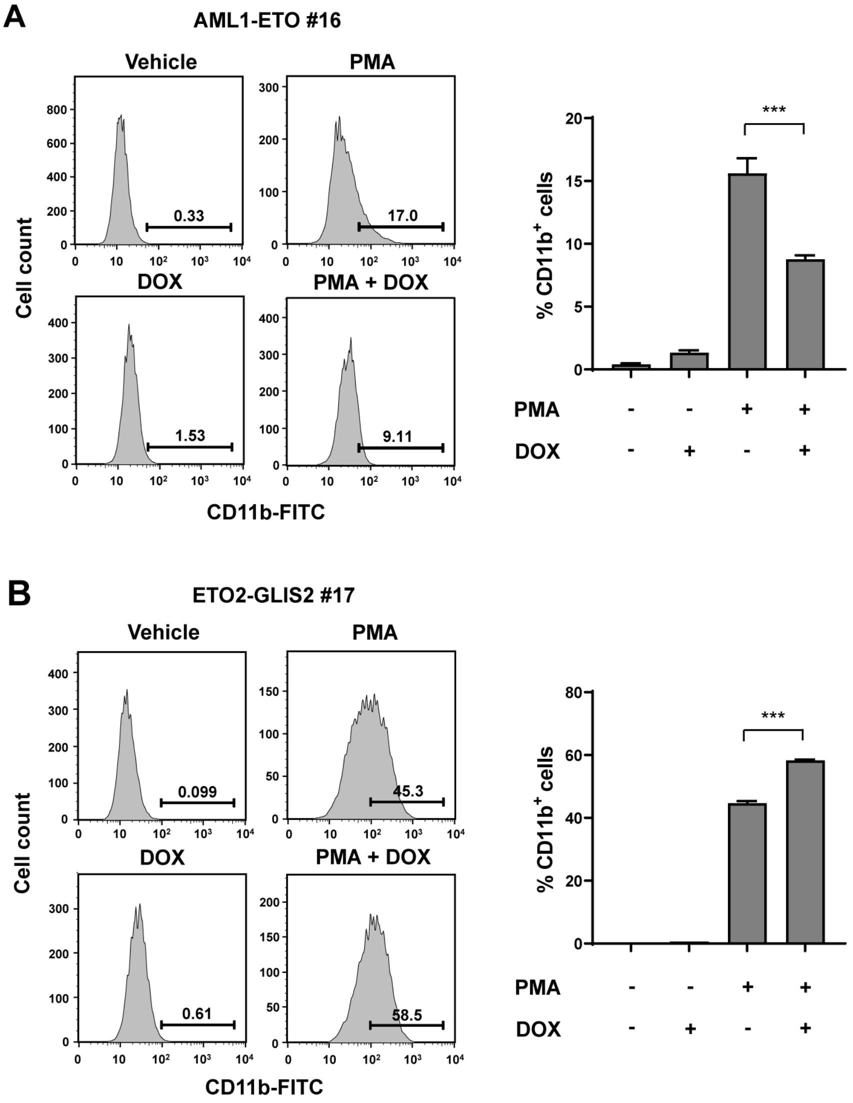

Supplementary Figure S1. Myeloid differentiation analysis of extra cell lines of AML1-ETO (#16) and ETO2-GLIS2 (#17). **(A)** AML1-ETO inhibits myeloid differentiation. **(B)** ETO2-GLIS2 promotes myeloid differentiation. Establishment of the #16 and #17 cell lines, their induced expression of AML1-ETO and ETO2-GLIS2, and their growth curves are shown in Figure 1. Note that these two cell lines show very similar behaviors as #4 and #14, respectively, compared with the results shown in Figure 6. Data are presented as means ± SD of three separate experiments; two-tailed t-test; ***P < 0.001.

Supplementary Table S1. Sequence statistics of the ChIP-seq and RNA-seq analyses

Samples	Number of reads	Mapping rate
ChIP-seq		
AML1-ETO DOX 12 h ChIP	49909314	79.62%
AML1-ETO DOX 12 h Input	38965485	95.95%
AML1-ETO DOX 48 h ChIP	53175744	86.75%
AML1-ETO DOX 48 h Input	47538635	95.20%
ETO2-GLIS2 DOX 12 h ChIP	45070328	66.74%
ETO2-GLIS2 DOX 12 h Input	37742045	94.68%
ETO2-GLIS2 DOX 48 h ChIP	28048510	71.01%
ETO2-GLIS2 DOX 48 h Input	41072209	94.60%
RNA-seq		
Empty vector control	21970487	93.25%
Empty vector DOX 12 h	21056722	92.50%
Empty vector DOX 48 h	21435915	92.58%
AML1-ETO control	22468227	93.27%
AML1-ETO DOX 12 h	21462135	92.45%
AML1-ETO DOX 48 h	21549294	93.20%
ETO2-GLIS2 control	24928655	91.57%
ETO2-GLIS2 DOX 12 h	24495146	91.27%
ETO2-GLIS2 DOX 48 h	22709793	92.32%
